# Malnutrition Is an Independent Risk Factor for Low Health-Related Quality of Life Among Centenarians

**DOI:** 10.3389/fmed.2021.729928

**Published:** 2021-09-24

**Authors:** Shanshan Yang, Shengshu Wang, Wei Liu, Ke Han, Wangping Jia, Miao Liu, Yao He

**Affiliations:** ^1^Department of Disease Prevention and Control, The 1^st^ Medical Center, Chinese PLA General Hospital, Beijing, China; ^2^State Key Laboratory of Kidney Disease, Beijing Key Laboratory of Aging and Geriatrics, The 2^nd^ Clinical Center, Chinese PLA General Hospital, Institute of Geriatrics, Beijing, China; ^3^Department of Healthcare, Agency for Offices Administration, Central Military Commission, Beijing, China; ^4^Department of Innovative Medicine, Chinese PLA General Hospital, Beijing, China; ^5^Department of Statistics and Epidemiology, Graduate School, Chinese PLA General Hospital, Beijing, China

**Keywords:** centenarians, quality of life, nutritional status, gender disparity, Chinese

## Abstract

**Background:** To explore the association and understand gender disparities between nutritional status and quality of life among centenarians.

**Methods:** It was a full-sample survey of centenarians conducted in Hainan that included a total of 1,002 eligible centenarians whose age had been verified. The Mini Nutritional Assessment – Short Form (MNA-SF) questionnaire and the EuroQol five dimensions visual analog scale (EQ-5D-VAS) were used to measure participants' nutritional status and quality of life, respectively.

**Findings:** In the 1002 centenarians (822 women and 180 men), 797 (79.5%) (79.5%) reported multimorbidity. The adjusted standardized β estimate association between the MNA-SF and EQ-5D scores was 0.508 in the complete sample. With reference to the normal nutrition group, the standardized β estimate of the association between EQ-5D score and nutritional status were −0.179 and −0.583 for the at risk of malnutrition and malnutrition groups, respectively (both P <0.001). Nutritional status significantly affected the five dimensions of quality of life, particularly mobility and self-care. Compared with the normal nutrition group, the malnutrition group had greater odds of low mobility [Odds ratio (OR)=23.15; 95% CI: 9.81–54.64] and low self-care (OR=24.58; 95% CI: 12.62–47.89). Among males, nutritional status was significantly associated with the usual activities and anxiety/depression dimensions after adjustment. Female participants had results similar to the general population.

**Interpretation:** Malnutrition and being at risk of malnutrition is prevalent among centenarians. Maintaining normal nutritional status is an important protective factor and should receive more attention to improve centenarians' quality of life.

## Introduction

An aging population is the inevitable result of social and economic development. For an ever-increasing aging population, successful aging (SA) is the guarantee of and cornerstone to achieving healthy social development. According to current worldwide research, achieving SA means that the elderly not only have no difficulties in daily life and physiological functions, but are also mentally and emotionally healthy: they are the bio-psycho-social healthy elderly (1). Quality of life (QoL) in the aging population is a comprehensive reflection of health-related factors such as physical health, mental state, degree of independence, social relations, and environmental factors; and has been receiving increased attention (2).

Malnutrition is considered a common risk factor for cardiovascular and cerebrovascular diseases, chronic obstructive pulmonary disease (COPD), and even death (3–5). Previous studies reported that malnutrition also has a direct adverse effect on the quality of life in patients by affecting their muscle quality (6, 7). In recent years, researchers have paid attention to the relationship between nutritional status and quality of life in the elderly, however, most of the studies focus on institutionalized older adults, and the sample size was small (8–10). There are few relevant investigations on the community elderly population with a large sample, especially among the oldest members of the community.

Therefore, this study aims to assess the impact of nutritional status on the quality of life in a large sample of Chinese centenarians (people aged 100 years and older) and analyze gender disparities in these associations.

## Methods

### Participants

Data for this study were obtained from the China Hainan Centenarian Cohort Study (CHCCS) (11), the centenarians survey with the largest sample size till date. It was a full-sample survey of centenarians conducted in Hainan Province, China between 2014 and 2016 that included a total of 1,002 eligible centenarians whose age had been verified. Trained investigators conducted household surveys to collect a variety of information from research subjects. Questionnaire-based interviews, anthropometric measurements (height, weight, waist circumference, hip circumference, calf circumference, blood pressure, etc.), and collection of fasting blood samples for biochemical index testing were completed by systematically trained local nurses (local language speakers that had barrier-free communication) in Hainan.

### Definitions and Assessment Criteria

Age was calculated by subtracting the date of birth from the survey date. The date of birth was verified using information obtained from participants' identity (ID) cards and that provided by the Civil Affairs Bureau and the Public Security Bureau. The term “centenarians” refers to subjects who were 100 years of age or older at the time of the survey. Height and weight were measured by local trained nurses in Hainan using a physician weighing scale for the human body (11). The elderly interviewees were required to take off their shoes, caps, and coats; and keep aside personal belongings such as keys and mobile phones while the measurements were being taken. Height was rounded to the nearest 0.5 cm, and an error of <0.5 kg was permitted between two consecutive weight measurements. Body Mass Index (BMI) was calculated by dividing the weight in kilograms by the square of the height in meters. Multimorbidity was defined as having two or more chronic diseases simultaneously (12).

The nutritional status of the centenarians was evaluated using the Mini Nutritional Assessment—Short Form (MNA-SF) questionnaire (Chinese validated versions), consisting of six components: appetite loss, weight loss, mobility, stress/acute disease, dementia/depression, and BMI/CC (13). Out of a total of 14 possible points, a score of 12–14 points indicated good nutritional status, 8–11 points indicated being at risk of malnutrition, and ≤ 7 points indicated malnutrition. Higher individual and overall MNA-SF scores indicated better nutritional status.

Quality of life was measured using the EuroQol five dimensions visual analog scale (EQ-5D-VAS) instrument (Chinese validated versions) (14). The EQ-5D is a widely used, validated questionnaire that includes five health dimensions (mobility, self-care, daily activities, pain/discomfort, and anxiety/depression); and each dimension contains three levels: no difficulty, some difficulties, and extreme difficulties. The Visual Analogue Scale (VAS) is a self-assessment tool where respondents self-assess their own health status on a 20 cm vertical scale. The top 100 points indicate the best health status, whereas the bottom zero points indicate the worst health status. Systematically trained local nurses in Hainan conducted interviews and collected information on EQ-5D and VAS, and used the Japanese population time trade-off (TTO) model to calculate the health indices, since previous studies had found that this model was currently the most suitable tool for use in Chinese people (15, 16). The scores generated by this conversion table ranged from −0.11 to 1.00. An EQ-5D abnormality with a score of <1 in any dimension was defined as low EQ-5D score. If the score based on the five dimensions of mobility, self-care, daily activities, pain/discomfort, and no difficulty level, then the quality of life dimension was defined as normal score; if not, it was defined as low score.

The covariates included in this study comprise gender, ethnicity (Han, Li, and other minorities), marital status (married, widowed, and others), educational level (illiterate, elementary, and middle school and above), residential type (living with family and living alone), smoking status (non-smoker, previous smoker, and current smoker), drinking status (non-drinker, previous drinker, and current drinker) and physical activity level (low, middle, and high). Multimorbidity was defined as patients living with two or more chronic health conditions (17). Data were collected using household surveys conducted by trained Hainan local nurses who were local language speakers and had barrier-free communication.

### Statistical Analyses

The research data were assembled into the software database Epidata 3.1 using double entry. The variable representing nutritional status was operationalized as both total MNA-SF score and as a three-level categorical variable. Continuous variables were described in terms of mean ± standard deviation, and differences between nutritional status groups were compared using the independent sample *t* test. Categorical variables were described in terms of frequencies and percentages [*n* (%)], and the chi-square test was used to compare group differences. In addition, univariate and multivariate logistic and linear regression models were used to analyze the association between nutritional status and quality of life (measured by the EQ-5D-VAS score). The MNA-SF score (the higher the score, the better the nutritional status) and the three nutritional status groups (considering normal nutritional status as the reference group) were added as independent variables to the linear regression model that adjusted for demographic characteristics (gender, age, ethnicity, marital status, education levels, and residential type) and lifestyle variables (smoking, drinking, and physical activity). Each test of hypothesis was two-sided, with *P* <0.05 as the cutoff for statistical significance. All statistical analyses were performed using the SPSS 22.0 software package.

### Ethics

The CHCCS was conducted in accordance with the Declaration of Helsinki and was approved by the Medical Ethics Committee of the Chinese PLA General Hospital (301hn11-206-01). All participants provided written informed consent before joining the study.

## Results

### Description of Participant Characteristics

The study included 1,002 centenarians, comprising of 822 women and 180 men, with an average age of 102.77 ± 2.75 years, of which 797 (79.5%) reported multimorbidity. Participants scored an average of 9.22 ± 2.06 on the MNA-SF scale; of which 123 (12.28%) had normal nutritional status, 671 (66.97%) were at risk of malnutrition, and 208 (20.76%) had malnutrition. Statistically significant differences existed among nutritional status groups in the distribution of gender, age, education level, physical activity, and continuous and categorical quality of life indicators (Table 1, *P* <0.05). The TTO-transformed a mean score of 0.62 ± 0.25; the average VAS score was 61.60 ± 15.56. There was a correlation between these two scores (correlation coefficient = 0.414, *P* <0.01) that differed significantly by gender.

**Table 1:** Sociodemographic characteristics of the CHCCS participants.

	**All (*n* = 1,002)**	**Normal nutritional status (*n* = 123)**	**At risk of malnutrition (*n* = 671)**	**Malnutrition (*n* = 208)**	***P***
**Continuous variables (Mean ± SD)**					
Age (years)	102.78 ± 2.73	102.53 ± 2.62	102.79 ± 2.75	102.88 ± 2.72	0.505
MNA-SF	9.22 ± 2.06	12.5 ± 0.74	9.53 ± 1.11	6.27 ± 0.92	<0.001
EQ5D	0.62 ± 0.25	0.77 ± 0.17	0.66 ± 0.21	0.38 ± 0.25	<0.001
VAS	61.60 ± 15.56	67.57 ± 12.67	63.31 ± 13.93	52.55 ± 18.32	<0.001
**Categorical variables [** ***N*** **(%)]**					
Gender					0.008
Male	180 (18.0)	30 (16.7)	126 (70)	24 (13.3)	
Female	822 (82.0)	93 (11.3)	545 (66.3)	184 (22.4)	
Age group					0.031
100–104	795 (79.3)	109 (13.7)	526 (66.2)	160 (20.1)	
105–109	176 (17.6)	9 (5.1)	125 (71)	42 (23.9)	
≥110	31 (3.1)	5 (16.1)	20 (64.5)	6 (19.4)	
Ethnic					0.609
Han	883 (88.1)	111 (12.6)	590 (66.8)	182 (20.6)	
Li	106 (10.6)	11 (10.4)	70 (66)	25 (23.6)	
Others	13 (1.3)	1 (7.7)	11 (84.6)	1 (7.7)	
Marital status					0.661
Married	100 (10.0)	14 (14)	67 (67)	19 (19)	
Widowed	836 (83.4)	104 (12.4)	560 (67)	172 (20.6)	
Divorced or never married	66 (6.6)	5 (7.6)	44 (66.7)	17 (25.8)	
Education level					0.013
Illiterate	915 (91.3)	104 (11.4)	617 (67.4)	194 (21.2)	
Primary school	67 (6.7)	12 (17.9)	43 (64.2)	12 (17.9)	
Middle school or higher	20 (2.0)	7 (35)	11 (55)	2 (10)	
Residential type					0.004
Living together with families	863 (86.1)	104 (12.1)	565 (65.5)	194 (22.5)	
Living alone at home	139 (13.9)	19 (13.7)	106 (76.3)	14 (10.1)	
Smoking status					0.744
Non-smoker	919 (91.7)	112 (12.2)	615 (66.9)	192 (20.9)	
Former	52 (5.2)	5 (9.6)	37 (71.2)	10 (19.2)	
Current	31 (3.1)	6 (19.4)	19 (61.3)	6 (19.4)	
Drinking status					0.276
Non-drinker	872 (87.0)	107 (12.3)	584 (67)	181 (20.8)	
Former	81 (8.1)	6 (7.4)	56 (69.1)	19 (23.5)	
Current	49 (4.9)	10 (20.4)	31 (63.3)	8 (16.3)	
Physical activity					<0.001
Low	874 (87.2)	100 (11.4)	579 (66.2)	195 (22.3)	
Medium	37 (3.7)	12 (32.4)	22 (59.5)	3 (8.1)	
High	91 (9.1)	11 (12.1)	70 (76.9)	10 (11)	
EQ5D					<0.001
High QOL	129 (12.9)	35 (27.1)	90 (69.8)	4 (3.1)	
Low QOL	873 (87.1)	88 (10.1)	581 (66.6)	204 (23.4)	

### Association of Nutritional Status and Quality of Life

#### Taking EQ-5D Score as the Dependent Variable

After adjusting for demographic characteristics and lifestyle-related variables, the standardized β estimate for the association of the MNA-SF scale score with the EQ-5D score in the complete sample was 0.508 (*P* < 0.001). Compared with the normal nutritional status group, the standardized β estimates for the association with EQ-5D in the at risk of malnutrition and malnutrition groups were −0.179 and −0.583, respectively (both *P* <0.001, Table 2). After adjustment for potential confounders, the three standardized β were 0.357, −0.151, and −0.394 among males (Supplementary Table 1); and 0.530, −0.189, and −0.620 among females (Supplementary Table 2, *P* <0.001).

**Table 2:** Association between nutritional status and quality of life (EQ-5D) scores.

		**95% CI**			
**Model A**	**β**	**lower**	**upper**	**Standard β**	***P***
MNA-SF (continuous)	0.065	0.059	0.071	0.538	0.000
**Normal nutritional status (reference)**					
At risk of malnutrition	−0.104	−0.146	−0.063	−0.197	0.000
Malnutrition	−0.383	−0.431	−0.335	−0.624	0.000
**Model B**					
MNA-SF (continuous)	0.064	0.058	0.070	0.532	0.000
**Normal nutritional status (reference)**					
At risk of malnutrition	−0.101	−0.143	−0.060	−0.192	0.000
Malnutrition	−0.377	−0.425	−0.328	−0.614	0.000
**Model C**					
MNA-SF (continuous)	0.064	0.057	0.070	0.530	0.000
Normal nutritional status (reference)					
At risk of malnutrition	−0.097	−0.139	−0.055	−0.184	0.000
Malnutrition	−0.373	−0.421	−0.324	−0.608	0.000
**Model D**					
MNA-SF (continuous)	0.061	0.055	0.067	0.508	0.000
**Normal nutritional status (reference)**					
At risk of malnutrition	−0.094	−0.134	−0.055	−0.179	0.000
Malnutrition	−0.357	−0.404	−0.311	−0.583	0.000

*The score of MNA-SF (continuous) and the classification of nutritional risk (categorical) were involved in the models separately*.

*Model A: Crude model*.

*Model B: Adjusted for gender, age*.

*Model C: Adjusted for gender, age, ethnic, education level, residential type*.

*Model D: Adjusted for gender, age, ethnic, education level, residential type, smoking, drinking, physical activity*.

#### Taking VAS as the Dependent Variable

After adjusting for demographic characteristics and lifestyle-related variables, the standardized β estimate for the association of the MNA-SF scale score with the VAS score in the complete sample was 0.319 (*P* <0.001). Compared with the normal nutritional status group, the standardized β estimates for the VAS score in the at risk of malnutrition and malnutrition groups were −0.106 and −0.354, respectively (both *P* <0.05, Table 3). for potential confounders, the three standardized β estimates were 0.237, −0.092, and −0.292 among male participants (Supplementary Table 3); and 0.336, −0.112, and −0.374 among female participants (Supplementary Table 4, *P* <0.05).

**Table 3:** Association between nutritional status and quality of life (VAS) scores.

		**95% CI**			
**Model A**	**β**	**Lower**	**Upper**	**Standard β**	***P***
MNA-SF (continuous)	2.605	2.166	3.044	0.346	0.000
**Normal nutritional status (reference)**					
At risk of malnutrition	−4.262	−7.112	−1.412	−0.129	0.003
Malnutrition	−15.016	−18.322	−11.711	−0.392	0.000
**Model B**					
MNA-SF (continuous)	2.525	2.082	2.968	0.335	0.000
**Normal nutritional status (reference)**					
At risk of malnutrition	−3.917	−6.762	−1.071	−0.118	0.007
Malnutrition	−14.416	−17.726	−11.107	−0.376	0.000
**Model C**					
MNA-SF (continuous)	2.504	2.062	2.946	0.332	0.000
**Normal nutritional status (reference)**					
At risk of malnutrition	−3.563	−6.412	−0.713	−0.108	0.014
Malnutrition	−14.100	−17.410	−10.790	−0.368	0.000
**Model D**					
MNA-SF (continuous)	2.403	1.964	2.842	0.319	0.000
**Normal nutritional status (reference)**					
At risk of malnutrition	−3.503	−6.320	−0.687	−0.106	0.015
Malnutrition	−13.576	−16.856	−10.296	−0.354	0.000

*The score of MNA-SF (continuous) and the classification of nutritional risk (categorical) were involved in the models separately*.

*Model A: Crude model*.

*Model B: Adjusted for gender, age*.

*Model C: Adjusted for gender, age, ethnic, education level, residential type*.

*Model D: Adjusted for gender, age, ethnic, education level, residential type, smoking, drinking, physical activity*.

### Impact of Nutrition on Quality of Life

Among the 1,002 centenarians studied, 873 had low EQ-5D score, including 143 men and 730 women. Logistic regression analysis using a binary outcome variable for high and low EQ-5D score found that the risk of having low EQ-5D score among centenarians decreases significantly as the MNA-SF score increases after adjusting for demographic characteristics (gender, age, ethnicity, marital status, education level, and residential type) and lifestyle factors (smoking status, drinking status, and physical activity); with an odds ratio (OR) of 0.69 (95% CI: 0.61–0.77, *P* < 0.001). Compared with the normal nutrition group, the risk of having low EQ-5D score was significantly higher in the at risk of malnutrition group (OR = 2.48, 95% CI: 1.53–4.03) and the malnutrition group (OR = 16.95, 95% CI: 5.72–50.26) respectively, after adjustment for potential confounders (P for trend < 0.001, Table 4). Results also showed that nutritional status had a statistically significant association with the five dimensions of quality of life after adjustment for potential confounders, of which mobility and self-care were impacted the most. Compared with the normal nutritional status group, the odds of low mobility and low self-care in the malnutrition group were 23.15 times (95% CI: 9.81–54.64) and 24.58 times (95% CI: 12.62–47.89), respectively (*P* < 0.001, Table 4). Among males, after adjustment for potential confounders, the nutritional status had a statistically significant association with only the usual activities and anxiety/depression dimensions of the quality of life scale compared with the normal nutrition group. The odds of low usual activities and anxiety/depression in the malnutrition group were 20.84 times (95% CI: 4.59–94.65) and 12.93 times (95% CI: 1.32–126.36) respectively, (*P* < 0.001, Supplementary Table 5). The results for female participants were similar to those for the entire study sample (Supplementary Table 6).

**Table 4:** The odds ratios (95%CI) of nutritional status on low EQ-5D score and its sub-domains among centenarians.

	**Model A**	**Model B**	**Model C**	**Model D**
**Low EQ-5D score**				
Normal nutritional status (reference)				
At risk of malnutrition	2.57 (1.64–4.03)	2.44 (1.55–3.85)	2.34 (1.48–3.71)	2.48 (1.53–4.03)
Malnutrition	20.28 (7.00–58.8)	18.50 (6.36–53.78)	17.72 (6.08–51.65)	16.95 (5.72–50.26)
P for trend	<0.001	<0.001	<0.001	<0.001
**Low mobility**				
Normal nutritional status (reference)				
At risk of malnutrition	2.48 (1.67–3.69)	2.40 (1.61–3.57)	2.40 (1.61–3.59)	2.54 (1.67–3.87)
Malnutrition	24.00 (10.43–55.2)	22.45 (9.74–51.75)	22.5 (9.74–51.97)	23.15 (9.81–54.64)
P for trend	<0.001	<0.001	<0.001	<0.001
**Low self-care**				
Normal nutritional status (reference)				
At risk of malnutrition	2.43 (1.62–3.65)	2.36 (1.57–3.55)	2.33 (1.55–3.52)	2.41 (1.57–3.69)
Malnutrition	21.9 (11.86–40.44)	20.87 (11.29–38.6)	20.65 (11.15–38.22)	24.58 (12.62–47.89)
P for trend	<0.001	<0.001	<0.001	<0.001
**Low usual activities**				
Normal nutritional status (reference)				
At risk of malnutrition	2.18 (1.47–3.24)	2.11 (1.41–3.14)	2.05 (1.37–3.06)	2.19 (1.42–3.36)
Malnutrition	14.02 (6.93–28.34)	13.07 (6.44–26.49)	12.73 (6.27–25.87)	13.6 (6.42–28.8)
P for trend	<0.001	<0.001	<0.001	<0.001
**Pain/discomfort**				
Normal nutritional status (reference)				
At risk of malnutrition	1.28 (0.87–1.9)	1.27 (0.86–1.88)	1.24 (0.83–1.83)	1.23 (0.83–1.83)
Malnutrition	2.79 (1.76–4.42)	2.74 (1.73–4.35)	2.68 (1.68–4.26)	2.58 (1.62–4.12)
P for trend	<0.001	<0.001	<0.001	<0.001
**Anxiety/depression**				
Normal nutritional status (reference)				
At risk of malnutrition	2.18 (0.98–4.84)	2.23 (1–4.95)	2.15 (0.97–4.81)	2.11 (0.94–4.72)
Malnutrition	8.05 (3.56–18.2)	8.28 (3.65–18.79)	8.07 (3.55–18.36)	7.42 (3.25–16.96)
P for trend	<0.001	<0.001	<0.001	<0.001

*Model A: Crude model*.

*Model B: Adjusted for gender, age*.

*Model C: Adjusted for gender, age, ethnic, education level, residential type*.

*Model D: Adjusted for gender, age, ethnic, education level, residential type, smoking, drinking, physical activity*.

In the CHCCS, the prevalence of multimorbidity is 79.5% (797/1002), 85.6% in men and 78.2% in women. The most common diseases in the centenarians were hypertension and chronic kidney disease (CKD). When logistic regression analyses were stratified by multimorbidity, the MNA-SF score was included as a continuous variable in the model. After adjustment for potential confounders, the results showed that among the multimorbidity group, higher nutritional status score was a protective factor for overall quality of life and its five dimensions, with the following adjusted odds ratios (OR = 0.68, 95% CI: 0.60–0.77) for low QoL, (OR = 0.66, 95% CI: 0.60–0.73) for low mobility, (OR = 0.62, 95% CI: 0.56–0.68) for low self-care, usual activities, (OR = 0.82, 95% CI: 0.77–0.88) for pain/discomfort, and (OR = 0.72, 95% CI: 0.65–0.80) for anxiety/depression (all *P* < 0.05, Supplementary Table 7). In the non-multimorbidity group, higher nutritional status score was a protective factor for overall quality of life in the adjusted model (OR = 0.69, 95% CI: 0.52–0.92), but no statistically significant association was detected between nutritional status and the pain/discomfort dimension (Supplementary Table 7).

## Discussion

This study found that malnutrition and being at risk of malnutrition were very common in a large sample of centenarians, with only 12.3% having normal nutritional status. The correlation analysis showed that good nutritional status was a protective factor for the quality of life in centenarians, and would impact all its dimensions. While the mobility and self-care dimensions were most affected among females, the impact of nutritional status on quality of life was more prominently manifested in the form of low usual activities and anxiety/depression among males. Analyses stratified by multimorbidity found no significant difference in the association between nutritional status and quality of life between the two groups.

In the EQ-5D (self-reported version), VAS was retained to provide Supplementary Information to assess interviewees' health status (18). The results of the fifth national health service survey in China in 2013 showed that the correlation coefficient between EQ-5D and VAS was 0.529 in the general population and 0.414 in a sample of centenarians (19). A survey based on the British population showed that the coefficient was 0.51 among females (20). This difference may be due to different population age structure and social culture. A study showed that the mean EQ-5D index was 78.3 ± 15.8 among community members over 72 years of age in Germany; they used the original value from 0 to 100, and the EQ-5D index calculated using TTO conversion was not reported in the study (21). The mean VAS in this population was 61.60 ± 15.56, higher than the results of a previous survey focusing on elderly people with cognitive impairment. Among this population, 12.9% people had a good quality of life (EQ-5D score equal to 1), which was also higher than the proportion of elderly people with cognitive impairment (6.1%) (22). The profile reflected that the centenarians were relatively healthy. A population-based review of MNA-SF scores showed that the prevalence of being at risk of malnutrition (score ≤ 11 points) was 8–76% based on a nutritional survey conducted among the elderly in the community (23). A nutritional survey conducted among Japanese centenarians found that the proportion of being malnutrition was 34.7% (24). While the prevalence of being at risk of malnutrition in this population has reached 87.6%, it warrants more attention.

Previous studies of the association between nutritional status and quality of life have focused on either children and adolescents or elderly patients, including diabetics and renal dialysis patients (6, 25, 26), suggesting that good nutritional status was a protective factor for quality of life in these populations. In recent years, researchers have paid attention to the relationship between nutritional status and quality of life in the elderly, however, most of the studies focus on institutionalized older adults, and the sample size was small (8–10). There are few relevant investigations on the community elderly population with a large sample, especially among the oldest members of the community. One study on 102 elderly people over 75 years of age belonging to the Garrucha (Almería) community in southern Spain was found (27). The results showed that the risk of malnutrition was significantly negatively associated with quality of life indices (*P* < 0.05), similar to the conclusions from our study. However, the sample size of this study was small, the association had not been adjusted for other factors, and the impact of nutritional status on different sub-dimensions of quality of life had not been analyzed. One study on 9,996 inpatients of six tertiary-level hospitals in China (28) showed that 9.7% of the inpatients were malnourished, and higher MNA-SF scores were related to higher HRQoL scores in older patients. Only 256 (3%) of the study participants were 85 ys or older and they all institutionalized elderly population.

This study has certain limitations: First, this study used a cross-sectional study design, and therefore causal inferences cannot be drawn from the results. Second, the study population was a large sample of centenarians in an island environment, and caution should be used while generalizing the conclusions to other populations. Third, most of the information on the EQ-5D and MNA-SF scales were collected using self-reported questionnaires. There may be potential recall bias; however, uniformly trained investigators attempted to minimize this by confirming the collected information via inquiries to the centenarians and their families.

Despite certain limitations, this study analyzed the association between nutritional status and quality of life using the largest sample of centenarians till date (11), and found that nutritional status is a protective factor for quality of life, and impacts all its sub-dimensions in centenarians. The nutritional status of men and women has different effects on each sub-dimension of quality of life. The study findings suggest that attention should be paid to their nutritional status to improve quality of life in the elderly.

## Data Availability Statement

The raw data supporting the conclusions of this article will be made available by the authors, without undue reservation.

## Ethics Statement

The CHCCS was conducted in accordance with the Declaration of Helsinki and was approved by the Medical Ethics Committee of the Chinese PLA General Hospital (301hn11-206-01). All participants provided written informed consent before joining the study. The patients/participants provided their written informed consent to participate in this study.

## Author Contributions

SY, SW, WL, ML, and YH contributed to data analysis and manuscript writing. SY, SW, WL, WJ, KH, ML, and YH contributed to study design and data collection. All authors contributed to manuscript revision and approval of final submission.

## Conflict of Interest

The authors declare that the research was conducted in the absence of any commercial or financial relationships that could be construed as a potential conflict of interest.

## Publisher's Note

All claims expressed in this article are solely those of the authors and do not necessarily represent those of their affiliated organizations, or those of the publisher, the editors and the reviewers. Any product that may be evaluated in this article, or claim that may be made by its manufacturer, is not guaranteed or endorsed by the publisher.
