# Supplementary material for: Malnutrition Is an Independent Risk Factor for Low Health-Related Quality of Life Among Centenarians
Source: Front Med (Lausanne). 2021 Sep 24;8:729928. doi: 10.3389/fmed.2021.729928 (PMC8498216; doi:10.3389/fmed.2021.729928)
Supplement: Supplementary file 1 [file Data_Sheet_1.PDF]

Table S1 Association between nutrition status and EQ-5D scores in males

		95% CI			
**Model A**	β	lower	upper	Standard β	P
MNA-SF (continuous)	0.050	0.033	0.067	0.395	0.000
Normal nutritional status (reference)					
At risk of malnutrition	-0.091	-0.182	-0.001	-0.174	0.047
Malnutrition	-0.322	-0.443	-0.200	-0.454	0.000
**Model B**					
MNA-SF (continuous)	0.050	0.033	0.067	0.396	0.000
Normal nutritional status (reference)					
At risk of malnutrition	-0.089	-0.180	0.003	-0.169	0.057
Malnutrition	-0.321	-0.443	-0.199	-0.453	0.000
**Model C**					
MNA-SF (continuous)	0.047	0.030	0.064	0.375	0.000
Normal nutritional status (reference)					
At risk of malnutrition	-0.080	-0.172	0.013	-0.152	0.091
Malnutrition	-0.301	-0.425	-0.177	-0.424	0.000
**Model D**					
MNA-SF (continuous)	0.045	0.028	0.062	0.357	0.000
Normal nutritional status (reference)					
At risk of malnutrition	-0.080	-0.168	0.009	-0.151	0.077
Malnutrition	-0.279	-0.399	-0.159	-0.394	0.000

The score of MNA-SF (continuous) and the classification of nutritional risk (categorical) were involved in the models separately.

Model A: crude model;

Model B: Adjusted for age;

Model C: Adjusted for age, ethnic, education level, residential type;

Model D: Adjusted for age, ethnic, education level, residential type, smoking, drinking, physical activity.

Table S2 Association between nutrition status and EQ-5D scores in females

Model A	β	95% CI lower	upper	Standard β	P
MNA-SF (continuous)	0.067	0.060	0.074	0.557	0.000
Normal nutritional status (reference)					
At risk of malnutrition	-0.106	-0.152	-0.059	-0.201	0.000
Malnutrition	-0.387	-0.440	-0.334	-0.650	0.000
**Model B**					
MNA-SF (continuous)	0.067	0.060	0.074	0.557	0.000
Normal nutritional status (reference)					
At risk of malnutrition	-0.105	-0.152	-0.058	-0.201	0.000
Malnutrition	-0.387	-0.440	-0.333	-0.650	0.000
**Model C**					
MNA-SF (continuous)	0.067	0.060	0.074	0.558	0.000
Normal nutritional status (reference)					
At risk of malnutrition	-0.103	-0.151	-0.056	-0.197	0.000
Malnutrition	-0.387	-0.440	-0.333	-0.649	0.000
**Model D**					
MNA-SF (continuous)	0.064	0.057	0.070	0.530	0.000
Normal nutritional status (reference)					
At risk of malnutrition	-0.099	-0.144	-0.054	-0.189	0.000
Malnutrition	-0.369	-0.420	-0.318	-0.620	0.000

The score of MNA-SF (continuous) and the classification of nutritional risk (categorical) were involved in the models separately.
Model A: crude model;
Model B: Adjusted for age;
Model C: Adjusted for age, ethnic, education level, residential type;
Model D: Adjusted for age, ethnic, education level, residential type, smoking, drinking, physical activity.

Table S3 Association between nutrition status and VAS in males

		95% CI			
**Model A**	β	lower	upper	Standard β	P
MNA-SF (continuous)	1.952	0.887	3.017	0.262	0.000
Normal nutritional status (reference)					
At risk of malnutrition	-4.163	-9.726	1.399	-0.133	0.141
Malnutrition	-14.308	-21.807	-6.810	-0.339	0.000
**Model B**					
MNA-SF (continuous)	2.013	0.971	3.056	0.270	0.000
Normal nutritional status (reference)					
At risk of malnutrition	-3.003	-8.495	2.488	-0.096	0.282
Malnutrition	-14.170	-21.503	-6.837	-0.336	0.000
**Model C**					
MNA-SF (continuous)	1.883	0.825	2.941	0.252	0.001
Normal nutritional status (reference)					
At risk of malnutrition	-2.826	-8.410	2.757	-0.090	0.319
Malnutrition	-13.458	-20.954	-5.962	-0.319	0.001
**Model D**					
MNA-SF (continuous)	1.765	0.700	2.831	0.237	0.001
Normal nutritional status (reference)					
At risk of malnutrition	-2.881	-8.427	2.665	-0.092	0.307
Malnutrition	-12.323	-19.850	-4.795	-0.292	0.001

The score of MNA-SF (continuous) and the classification of nutritional risk (categorical) were involved in the models separately.

Model A: crude model;

Model B: Adjusted for age;

Model C: Adjusted for age, ethnic, education level, residential type;

Model D: Adjusted for age, ethnic, education level, residential type, smoking, drinking, physical activity.

Table S4 Association between nutrition status and VAS in females

		95% CI			
**Model A**	β	lower	upper	Standard β	P
MNA-SF (continuous)	2.648	2.160	3.137	0.349	0.000
Normal nutritional status (reference)					
At risk of malnutrition	-4.054	-7.349	-0.758	-0.122	0.016
Malnutrition	-14.622	-18.359	-10.885	-0.388	0.000
**Model B**					
MNA-SF (continuous)	2.638	2.150	3.127	0.347	0.000
Normal nutritional status (reference)					
At risk of malnutrition	-3.974	-7.272	-0.675	-0.120	0.018
Malnutrition	-14.512	-18.254	-10.770	-0.385	0.000
**Model C**					
MNA-SF (continuous)	2.645	2.158	3.132	0.348	0.000
Normal nutritional status (reference)					
At risk of malnutrition	-3.760	-7.049	-0.471	-0.113	0.025
Malnutrition	-14.493	-18.220	-10.766	-0.385	0.000
**Model D**					
MNA-SF (continuous)	2.555	2.069	3.041	0.336	0.000
Normal nutritional status (reference)					
At risk of malnutrition	-3.716	-6.973	-0.459	-0.112	0.025
Malnutrition	-14.093	-17.797	-10.389	-0.374	0.000

The score of MNA-SF (continuous) and the classification of nutritional risk (categorical) were involved in the models separately.

Model A: crude model;

Model B: Adjusted for age;

Model C: Adjusted for age, ethnic, education level, residential type;

Model D: Adjusted for age, ethnic, education level, residential type, smoking, drinking, physical activity.

Table S5 The Odds Ratio (95% CI) of nutrition status on low QoL sub-domains in males

	Model A	Model B	Model C	Model D
**Low Mobility**				
MNA-SF (continuous variable)	0.85 (0.72-1)	0.84 (0.71-1)	0.85 (0.72-1.01)	0.84 (0.7-1.01)
Normal nutritional status (reference)	1	1	1	1
At risk of malnutrition	1.20 (0.53-2.71)	1.07 (0.47-2.46)	1.12 (0.48-2.61)	1.15 (0.48-2.77)
Malnutrition	4.67 (1.14-19.17)	4.66 (1.13-19.21)	4.8 (1.13-20.35)	4.66 (1.04-20.9)
P for trend	0.084	0.069	0.072	0.102
**Low Self-care**				
MNA-SF (continuous variable)	0.63 (0.52-0.76)	0.62 (0.51-0.75)	0.63 (0.52-0.76)	0.61 (0.49-0.75)
Normal nutritional status (reference)	1	1	1	1
At risk of malnutrition	3.87 (1.48-10.12)	3.61 (1.37-9.5)	3.69 (1.38-9.86)	4.04 (1.46-11.19)
Malnutrition	20 (4.94-80.89)	20.08 (4.95-81.4)	19.64 (4.73-81.63)	20.84 (4.59-94.65)
P for trend	<0.001	<0.001	<0.001	<0.001
**Low Usual Activities**				
MNA-SF (continuous variable)	0.76 (0.64-0.91)	0.76 (0.64-0.91)	0.77 (0.65-0.92)	0.76 (0.62-0.92)
Normal nutritional status (reference)	1	1	1	1
At risk of malnutrition	1.47 (0.66-3.28)	1.37 (0.61-3.09)	1.21 (0.53-2.78)	1.24 (0.51-3.03)
Malnutrition	9.62 (1.91-48.42)	9.61 (1.91-48.4)	8.25 (1.61-42.3)	7.92 (1.38-45.57)
P for trend	0.023	0.022	0.034	0.06
**Pain/discomfort**				
MNA-SF (continuous variable)	0.89 (0.77-1.04)	0.9 (0.77-1.05)	0.92 (0.78-1.08)	0.92 (0.78-1.09)
Normal nutritional status (reference)	1	1	1	1
At risk of malnutrition	1.16 (0.52-2.61)	1.23 (0.54-2.78)	1.08 (0.46-2.52)	1.09 (0.46-2.57)
Malnutrition	2.1 (0.7-6.26)	2.12 (0.71-6.32)	1.69 (0.55-5.23)	1.55 (0.49-4.9)

P for trend	0.354	0.374	0.583	0.719
**Anxiety/Depression**				
MNA-SF (continuous variable)	0.64 (0.5-0.82)	0.63 (0.49-0.81)	0.65 (0.49-0.85)	0.67 (0.51-0.89)
Normal nutritional status (reference)	1	1	1	1
At risk of malnutrition	3.62 (0.46-28.71)	3.39 (0.42-27.09)	3.21 (0.39-26.41)	3 (0.34-26.22)
Malnutrition	20.71 (2.41-178.24)	20.68 (2.4-178.11)	17.41 (1.93-156.93)	12.93 (1.32-126.36)
P for trend	0.001	<0.001	0.002	0.015

The score of MNA-SF (continuous) and the classification of nutritional risk (categorical) were involved in the models separately.

Model A: crude model;

Model B: Adjusted for age;

Model C: Adjusted for age, ethnic, education level, residential type;

Model D: Adjusted for age, ethnic, education level, residential type, smoking, drinking, physical activity.

Table S6 The Odds Ratio (95% CI) of nutrition status on low QoL sub-domains in females

	Model A	Model B	Model C	Model D
**Low Mobility**				
MNA-SF (continuous variable)	0.62 (0.56-0.69)	0.62 (0.56-0.69)	0.61 (0.55-0.68)	0.61 (0.54-0.68)
Normal nutritional status (reference)	1	1	1	1
At risk of malnutrition	3.05 (1.94-4.8)	3.03 (1.92-4.77)	3.07 (1.94-4.85)	3.29 (2.03-5.33)
Malnutrition	40.41 (13.84-117.94)	40.09 (13.73-117.06)	40.72 (13.92-119.15)	43.85 (14.5-132.67)
P for trend	<0.001	<0.001	<0.001	<0.001
**Low Self-care**				
MNA-SF (continuous variable)	0.61 (0.56-0.67)	0.61 (0.56-0.67)	0.61 (0.56-0.67)	0.6 (0.54-0.66)
Normal nutritional status (reference)	1	1	1	1
At risk of malnutrition	2.12 (1.35-3.35)	2.11 (1.34-3.32)	2.12 (1.35-3.35)	2.15 (1.34-3.47)
Malnutrition	21.07 (10.58-41.98)	20.87 (10.47-41.59)	20.94 (10.5-41.76)	25.28 (11.84-53.98)
P for trend	<0.001	<0.001	<0.001	<0.001
**Low Usural Activities**				
MNA-SF (continuous variable)	0.68 (0.62-0.75)	0.68 (0.62-0.75)	0.68 (0.61-0.74)	0.67 (0.61-0.75)
Normal nutritional status (reference)	1	1	1	1
At risk of malnutrition	2.41 (1.53-3.8)	2.39 (1.51-3.77)	2.39 (1.51-3.77)	2.57 (1.57-4.22)
Malnutrition	14.68 (6.69-32.2)	14.52 (6.61-31.87)	14.55 (6.62-31.97)	15.46 (6.66-35.89)
P for trend	<0.001	<0.001	<0.001	<0.001
**Pain/discomfort**				
MNA-SF (continuous variable)	0.82 (0.76-0.88)	0.82 (0.76-0.88)	0.82 (0.76-0.88)	0.82 (0.77-0.88)
Normal nutritional status (reference)	1	1	1	1
At risk of malnutrition	1.31 (0.84-2.05)	1.3 (0.83-2.04)	1.3 (0.83-2.05)	1.29 (0.82-2.03)
Malnutrition	2.91 (1.74-4.86)	2.88 (1.72-4.83)	2.89 (1.72-4.84)	2.78 (1.66-4.68)

P for trend	<0.001	<0.001	<0.001	<0.001
**Anxiety/Depression**				
MNA-SF (continuous variable)	0.72 (0.65-0.79)	0.72 (0.65-0.79)	0.72 (0.65-0.79)	0.73 (0.66-0.81)
Normal nutritional status (reference)	1	1	1	1
At risk of malnutrition	1.93 (0.81-4.59)	1.97 (0.83-4.69)	1.98 (0.83-4.73)	1.91 (0.8-4.58)
Malnutrition	6.67 (2.76-16.15)	6.89 (2.84-16.7)	6.92 (2.85-16.79)	6.3 (2.59-15.36)
P for trend	<0.001	<0.001	<0.001	<0.001

The score of MNA-SF (continuous) and the classification of nutritional risk (categorical) were involved in the models separately.

Model A: crude model;

Model B: Adjusted for age;

Model C: Adjusted for age, ethnic, education level, residential type;

Model D: Adjusted for age, ethnic, education level, residential type, smoking, drinking, physical activity.

Table S7 The Odds Ratio (95% CI) of nutrition status on low QoLand its sub-domains in multimorbidity group and without multimorbidity group

Multimorbidity	Model A	Model B	Model C	Model D
**Low QOL**	0.68 (0.60-0.76)	0.68 (0.61-0.77)	0.69 (0.61-0.77)	0.68 (0.60-0.77)
**Low Mobility**	0.67 (0.61-0.74)	0.68 (0.61-0.74)	0.67 (0.61-0.74)	0.66 (0.60-0.73)
**Low Self-care**	0.63 (0.58-0.69)	0.63 (0.58-0.69)	0.63 (0.58-0.69)	0.62 (0.56-0.68)
**Low Usural Activities**	0.70 (0.64-0.77)	0.71 (0.65-0.78)	0.71 (0.65-0.78)	0.69 (0.63-0.77)
**Pain/discomfort**	0.82 (0.76-0.88)	0.82 (0.76-0.88)	0.82 (0.76-0.88)	0.82 (0.77-0.88)
**Anxiety/Depression**	0.71 (0.65-0.79)	0.71 (0.64-0.79)	0.71 (0.64-0.79)	0.72 (0.65-0.80)
Without Multimorbidity				
**Low QOL**	0.67 (0.52-0.86)	0.69 (0.54-0.89)	0.68 (0.52-0.88)	0.69 (0.52-0.92)
**Low Mobility**	0.62 (0.50-0.76)	0.63 (0.51-0.78)	0.62 (0.50-0.77)	0.62 (0.49-0.79)
**Low Self-care**	0.52 (0.42-0.64)	0.52 (0.42-0.65)	0.52 (0.42-0.65)	0.49 (0.39-0.62)
**Low Usural Activities**	0.64 (0.52-0.78)	0.64 (0.53-0.79)	0.64 (0.52-0.78)	0.63 (0.51-0.79)
**Pain/discomfort**	0.87 (0.75-1.01)	0.87 (0.76-1.01)	0.87 (0.75-1.01)	0.88 (0.76-1.03)
**Anxiety/Depression**	0.71 (0.57-0.88)	0.70 (0.56-0.88)	0.70 (0.56-0.88)	0.69 (0.55-0.88)

Model A: crude model;

Model B: Adjusted for gender, age;

Model C: Adjusted for gender, age, ethnic, education level, residential type;

Model D: Adjusted for gender, age, ethnic, education level, residential type, smoking, drinking, physical activity.
